# Supplementary material for: mTORC1 accelerates retinal development via the immunoproteasome
Source: Nat Commun. 2018 Jun 27;9:2502. doi: 10.1038/s41467-018-04774-9 (PMC6021445; doi:10.1038/s41467-018-04774-9)
Supplement: Supplementary file 1 — Supplementary Information [file 41467_2018_4774_MOESM1_ESM.pdf]

*mTORC1 regulates retinal development via the immunoproteasome*

*Ji-Heon Choi et al.*

List of Supplementary Information

- Supplementary Figures 1 – 14
- Supplementary Tables 1 and 2

***Supplementary Fig. 1***

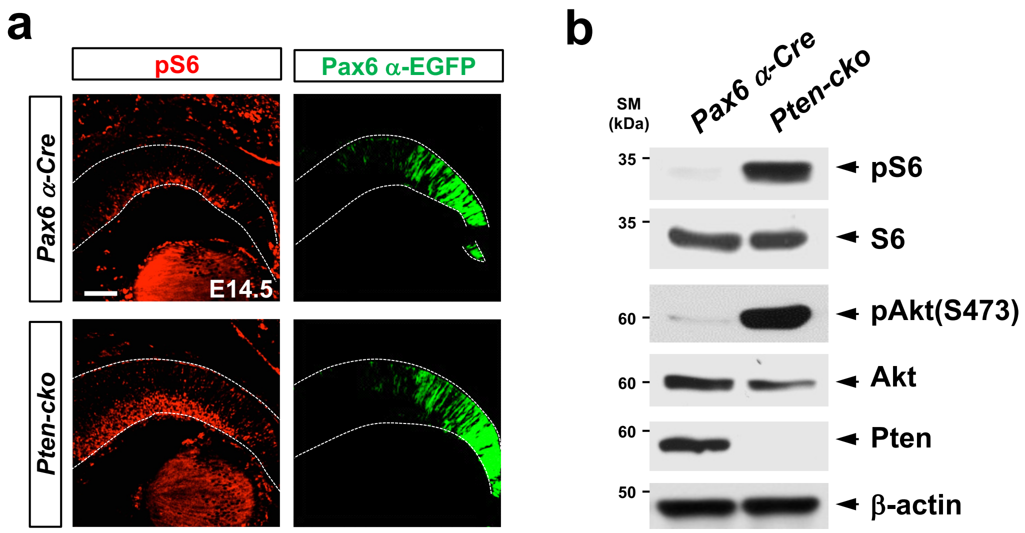


**Supplementary Figure 1. Activation of mTOR pathway in *Pten-cko* mouse retina.** (**a**) Distribution of cells having active mTORC1 in E14.5 *Pten$^{+/+}$;Pax6 α-Cre-IRES-EGFP* (*Pax6 α-Cre*) and *Pten$^{flox/flox}$;Pax6 α-Cre* (*Pten-cko*) littermate mouse retinas was investigated by immunostaining of the mouse retinal sections with anti-phospho-S6(Ser235/236) (pS6) antibody. Distribution of Pax6 α-enhancer-active RPCs in the retinas was also visualized by immunostaining of GFP, which is expressed under control of the α-enhancer. Dotted lines mark retinal boundaries. Scale bar denotes 100 μm. (**b**) Levels of pS6, S6, pAkt(S473), Akt, and Pten in E14.5 mouse retinal cell lysates were also examined by WB with the antibodies against corresponding proteins. Relative amount of proteins in each sample was determined by comparing β-actin levels of corresponding cell lysates.

***Supplementary Fig. 2***

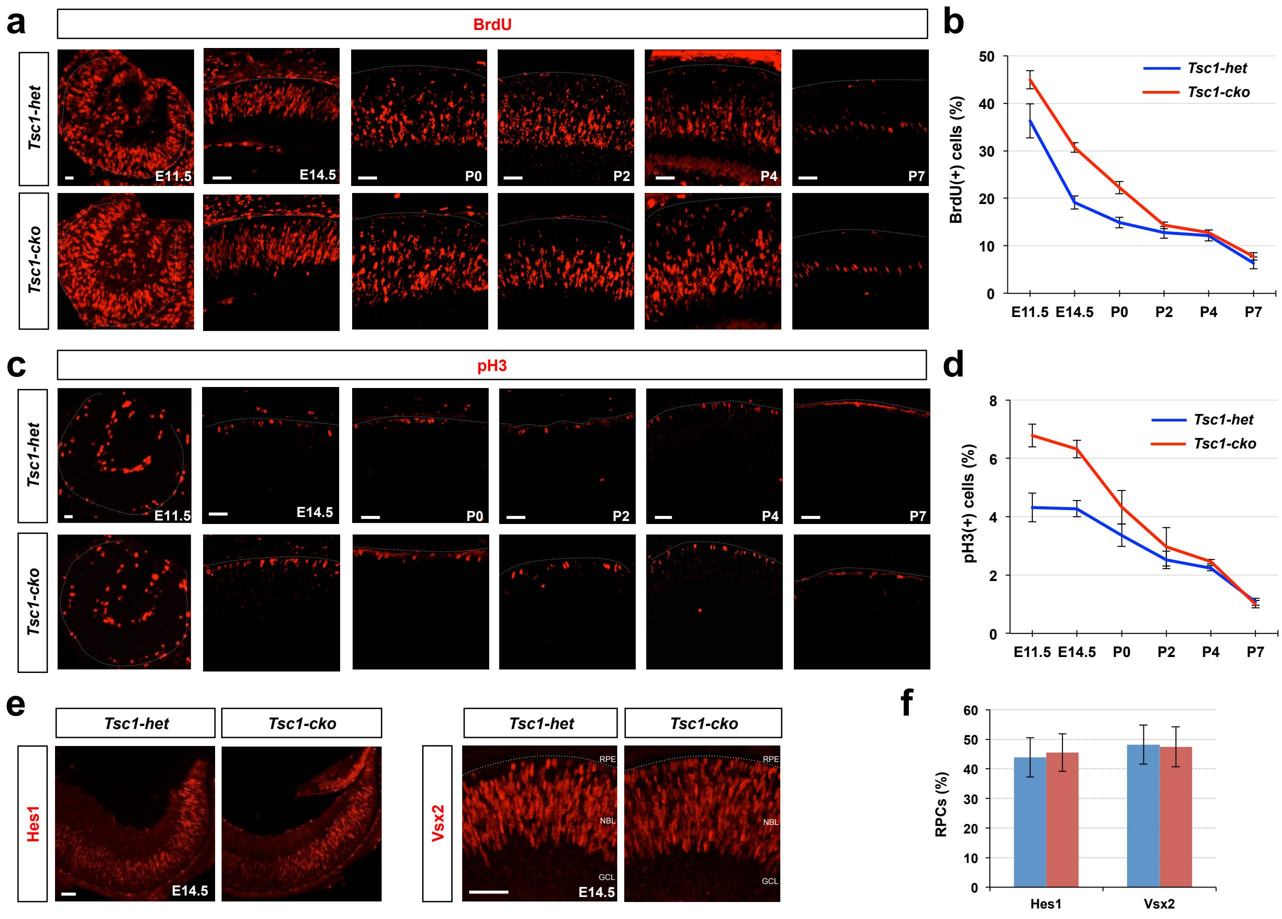


**Supplementary Figure 2. Hyperproliferation of RPCs in *Tsc1-cko* mouse retina.** (**a**) Proliferating cells in *Tsc1-het* and *Tsc1-cko* littermate mouse retinas at indicated developmental stages were detected by immunostaining of BrdU, which was provided by intraperitoneal injections to pregnant mice at 2h (E11.5; E14.5) or 3h (P0, P2, P4, P7) prior to sample collection. Scale bars, 50μm. (**c**) Mitotic RPCs were visualized by immunodetection of pH3. Scale bars, 50μm. Population of BrdU-positive proliferating cells (**b**) and pH3-positive mitotic cells (**d**) in the mouse retinas are shown in graphs. Numbers of retina analyzed are 5 (for E12.5, E14.5, and P0; 3 independent litters) or 6 (for P4 and P7; 4 independent litters). The y-axis values in the graph are average and error bars are SD. (**e**) The distribution of RPCs in E14.5 *Tsc1-het* and *Tsc1-cko* littermate mouse retinas was examined by immunostaining of RPC markers Hes1 and Vsx2. Scale bars, 100μm. (**f**) Hes1- and Vsx2-positive RPC populations in the mouse retinas are shown in a graph. The y-axis values in the graph are average and error bars are SD (n=4, 3 independent litters).

***Supplementary Fig. 3***

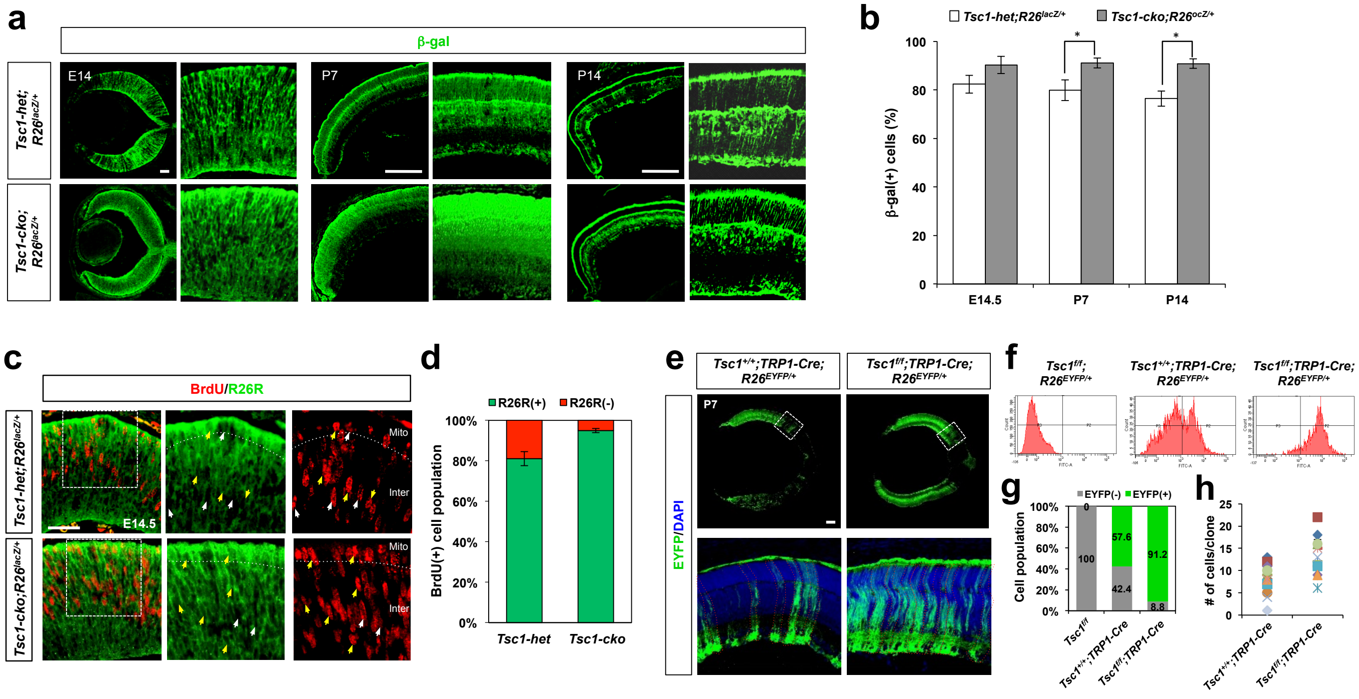


**Supplementary Figure 3. Rapid clonal expansion of *Tsc1*-defient RPC. (a)** Distribution of *Tsc1*-deficient cells in *Tsc1-cko* mouse retina is indirectly assessed by immunodetection of R26R β-gal expressed in *R26*$^{lacZ}$ loci after Cre-dependent excision of *loxP-STOP-loxP* cassette. **(b)** Average R26R-positive cell populations in the *Tsc1-het* and *Tsc1-cko* mouse retinas are shown in a graph. Numbers of retinas analyzed are 5 (for E14.5) or 4 (for P7 and P14) from 3 independent litters. *, *p*-value <0.05 (Student t-test). **(c)** Proliferating cells in the R26R-positive population, which are potential *Tsc1*-deficient cells in *Tsc1-cko* mouse retinas, and the R26R-negative population, which are WT cells in *Tsc1-het* and *Tsc1-cko* mouse retinas, were examined by immunodetection of BrdU incorporated into the retinal cells for 2h. (**d**) BrdU-positive cell populations, which are either positive or negative to R26R, are shown in a graph. The y-axis values in the graph are average and the error bars are SD. The numbers of retina analyzed are 4 from 3 independent litters. (**e**) Sections of P7 *Tsc1*$^{f/+}$*;TRP1-Cre;R26*$^{EYFP/+}$ and *Tsc1*$^{f/f}$*;TRP1-Cre;R26*$^{EYFP/+}$ littermate mouse eyes were immunostained with anti-GFP antibody to visualize R26-EYFP. Nuclei of the retinal cells were visualized by DAPI staining. (**f**) FACS profiles of P7 *Tsc1*$^{f/f}$*; R26*$^{EYFP/+}$, *Tsc1*$^{f/+}$*;TRP1-Cre;R26*$^{EYFP/+}$, and *Tsc1*$^{f/f}$*;TRP1-Cre;R26*$^{EYFP/+}$ littermate mouse retinal cells. Number of cells in each profile are 100,000. (**g**) R26-EYFP(-) and R26-EGFP(+) cell populations in each retinal sample are shown in a graph. The values in the y-axis are average (n=3, from 3 independent litters). (**h**) Numbers of cells in individual retinal clone (marked by red dotted area) were shown in a graph (n=15). Error bars denote SD. Scale bars in the pictures are 100μm.

***Supplementary Fig. 4***

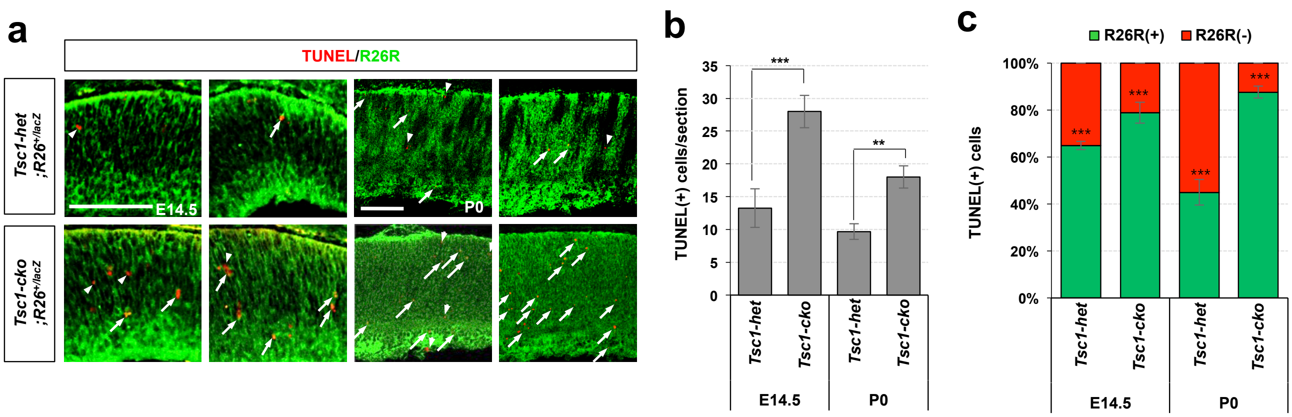


**Supplementary Figure 4. Enhanced apoptotic cell death of *Tsc1*-deficient cells.** (**a**) Apoptotic nuclei in E14.5 and P0 *Tsc1-het* and *Tsc1-cko* littermate mouse retinal sections were visualized by TUNEL staining (red) and subsequent immunodetection of R26R (green) expressed from $R26^{lacZ}$ loci. Arrows and arrowheads point to R26R-positive and R26-negative apoptotic cells, respectively. Scale bars denote 100 μm. (**b**) TUNEL-labeled cells in the sections were counted and the average numbers are shown in a graph. (**c**) R26R-negative and R26R-positive cell populations among total apoptotic cells are shown in a graph. The y-axis values of the graphs in (b) and (c) are averages and the numbers of sections analyzed are 8 (from 4 independent mouse eyes); error bars denote SD. **, *p*-value <0.01; ***, *p*-value <0.001 (Student t-test).

***Supplementary Fig. 5***

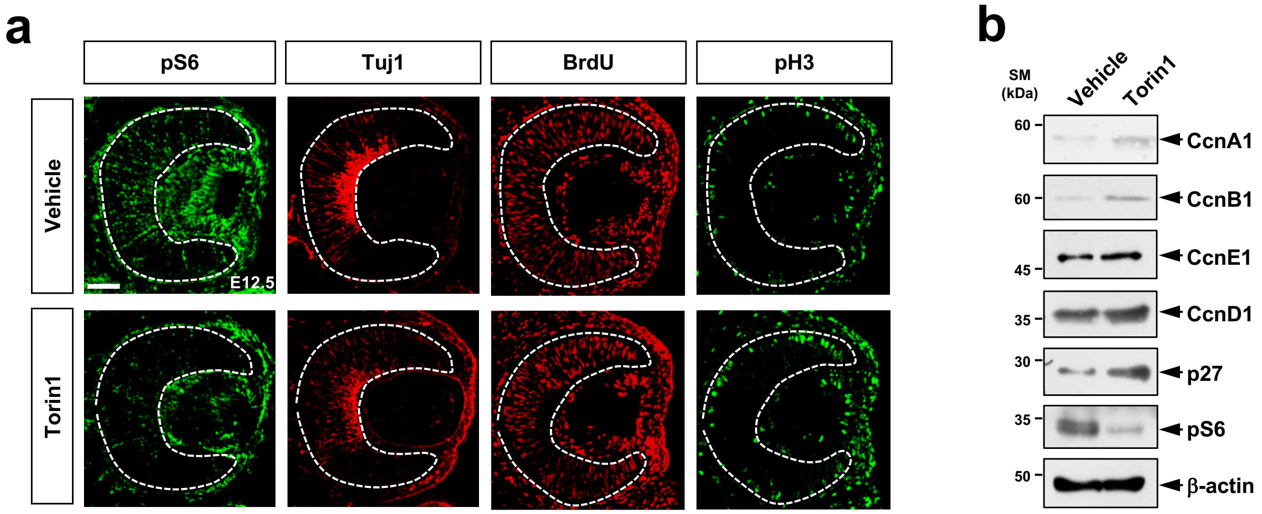


**Supplementary Figure 5. The inhibitory effects of Torin1 on mouse retinal development.** Pregnant mice were injected with vehicle or torin1 (5 μM) into the peritoneum at 12 dpc (a) and 14 dpc (b), and the embryos were collected after 12h. (**a**) BrdU was injected to the mice at 2h prior to the embryo collection. Distribution of each marker in E12.5 mouse embryonic retinas was investigated by immunostaining with corresponding antibodies. Scale bar, 100 μm. Dotted lines mark retinal boundaries. (**b**) Proteins (20 μg/lane) of cell lysates of E14.5 mouse retinas were analyzed by SDS-PAGE and subsequent WB analyses with the antibodies against corresponding proteins.

***Supplementary Fig. 6***

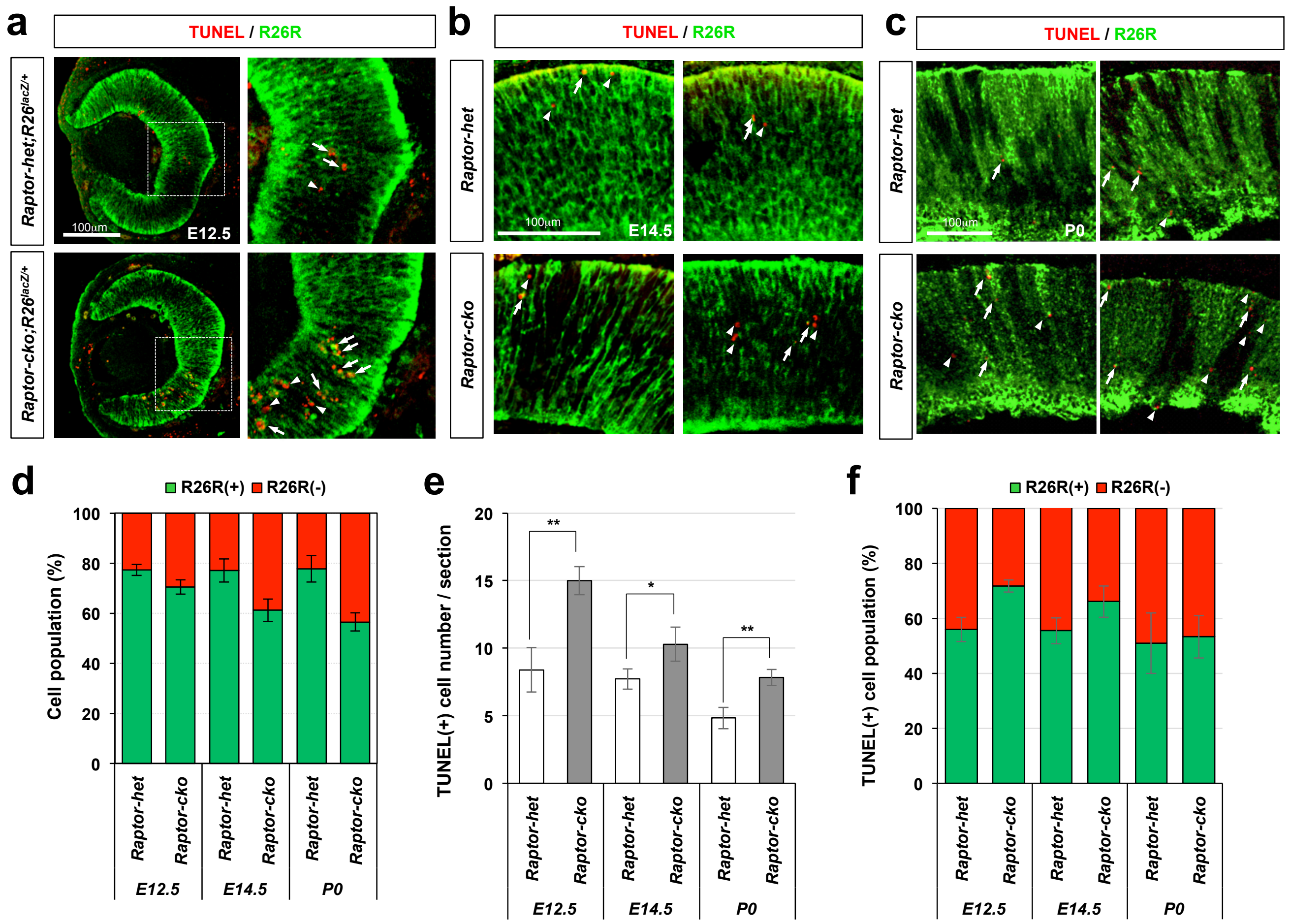


**Supplementary Figure 6. Gradual depletion of *Raptor*-deficient retinal cell population by apoptotic cell death.** The cells underwent Cre-dependent gene deletion in E12.5 (**a**), E14.5 (**b**), and P0 (**c**) littermate mouse retinas were visualized by immunodetection of R26R ß-gal (green). Apoptotic nuclei the mouse eye sections were also visualized by TUNEL staining (red). Arrows and arrowheads point to R26R-positive and R26-negative apoptotic cells, respectively. (**d**) R26R-positive and R26-negative cell populations in the retinas were quantified and shown in a graph. (**e**) TUNEL-labeled cells in the sections were counted and the average numbers are shown in a graph. (**f**) R26R-negative and R26R-positive cell populations among total apoptotic cells are shown in a graph. The y-axis values of the graphs in (d) – (f) are averages and the numbers of retinas analyzed are 6 (from 3 independent litters). Error bars denote SD. *, *p*-value <0.05; **, *p*-value <0.01 (Student t-test).

***Supplementary Fig. 7***

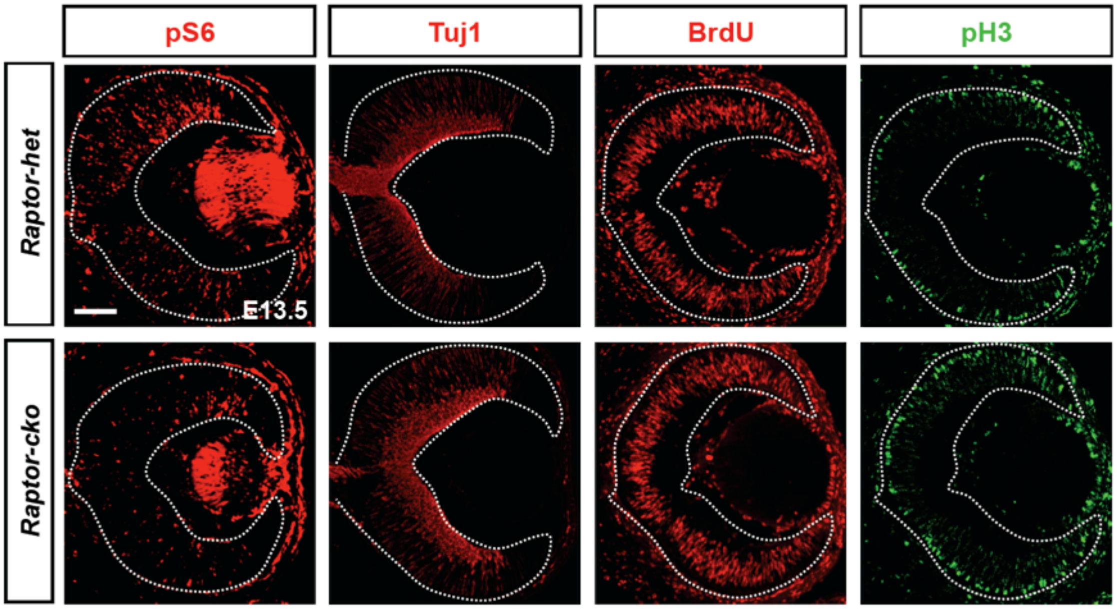


**Supplementary Figure 7. Anatomical analyses of *Raptor-cko* embryonic mouse retina.** E14.5 littermate mouse eye sections were stained with antibodies recognizing corresponding proteins. The quantifications of the results are shown in Fig.4f.

***Supplementary Fig. 8***

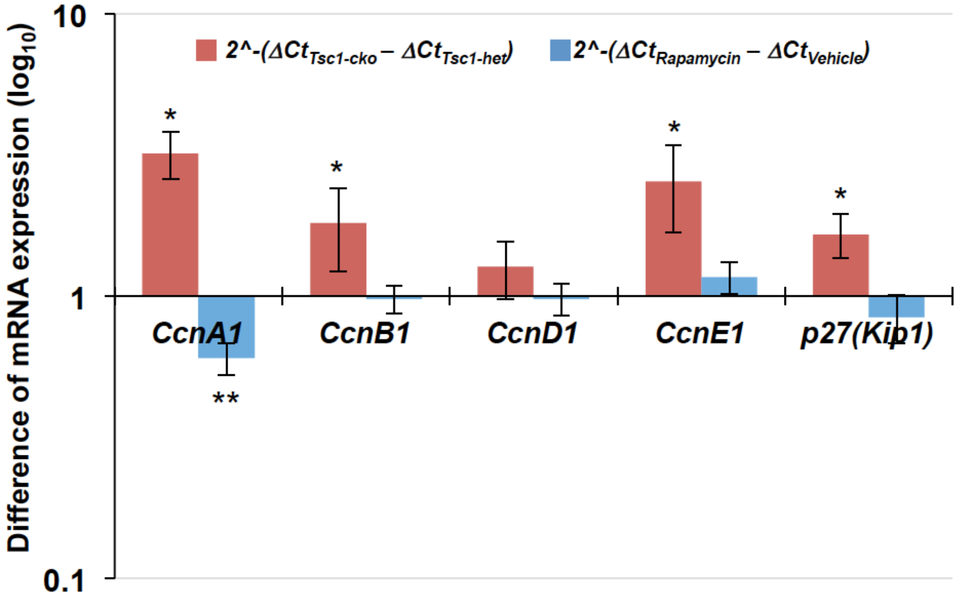


**Supplementary Figure 8. Expression of *cyclin* mRNAs in mouse retinas with altered mTORC1 activity.** Relative levels of *cyclin* and *p27* mRNA in E14.5 *Tsc1-het* and *Tsc1-cko* littermate mouse retinas were determined by RT-qPCR analyses with total RNA isolated from the mouse retinas. Alternatively, equal volume of rapamycin (5 mg/kg) or vehicle solution was injected into the intraperitoneal space of pregnant C56BL/6J female mice at 14 dpc and total RNA was isolated from the retina of E14.5 mouse embryos for RT-qPCR analyses. Average difference values of cycle threshold (ΔCt) of the corresponding genes were obtained by comparing with that of *β-actin* mRNA at same experimental batch. Relative expression levels of the genes were then obtained by comparing $2^{-\Delta\Delta Ct}$ values (see details in Experimental Procedures). Values are averages obtained from 5 independent experiments. Error bars denote SD. *, $p$-value < 0.05; **, $p$-value < 0.01 (Student t-test).

***Supplementary Fig. 9***

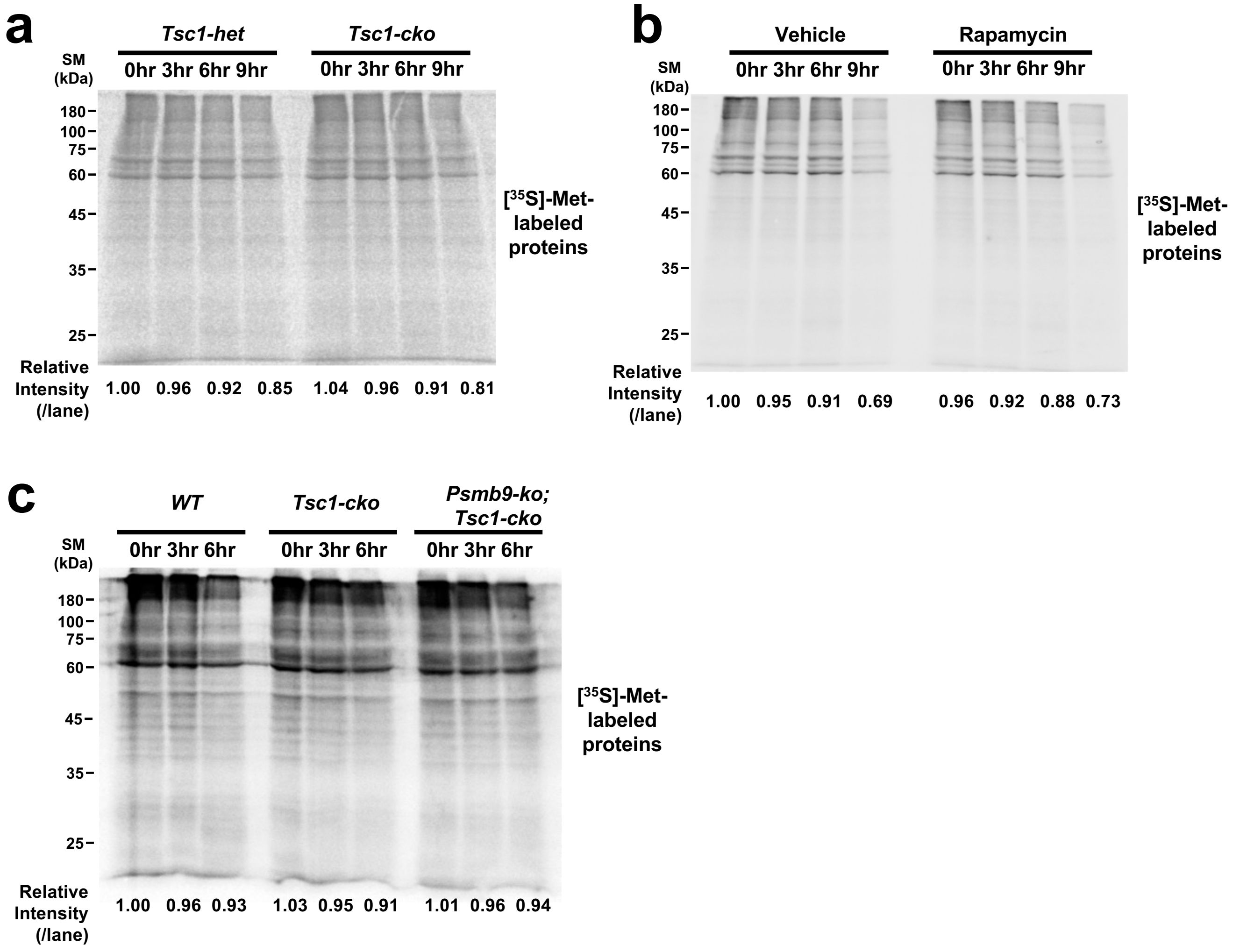


**Supplementary Figure 9. Total [$^{35}$S]-Met-labeled proteins in mouse retinas.** Cell lysates (20 μg/lane) of retinal explants, which were pulse-labeled and chased for the indicated time periods, were separated onto 10% SDS-PAGE and radioactive signals of emitted from the gels were detected by BAS7000 (Fuji). Experimental conditions were given in Fig.5e. The values below the autoradiogram images are relative intensity of [$^{35}$S]-Met-labeled proteins in each lane.

**_Supplementary Fig. 10_**

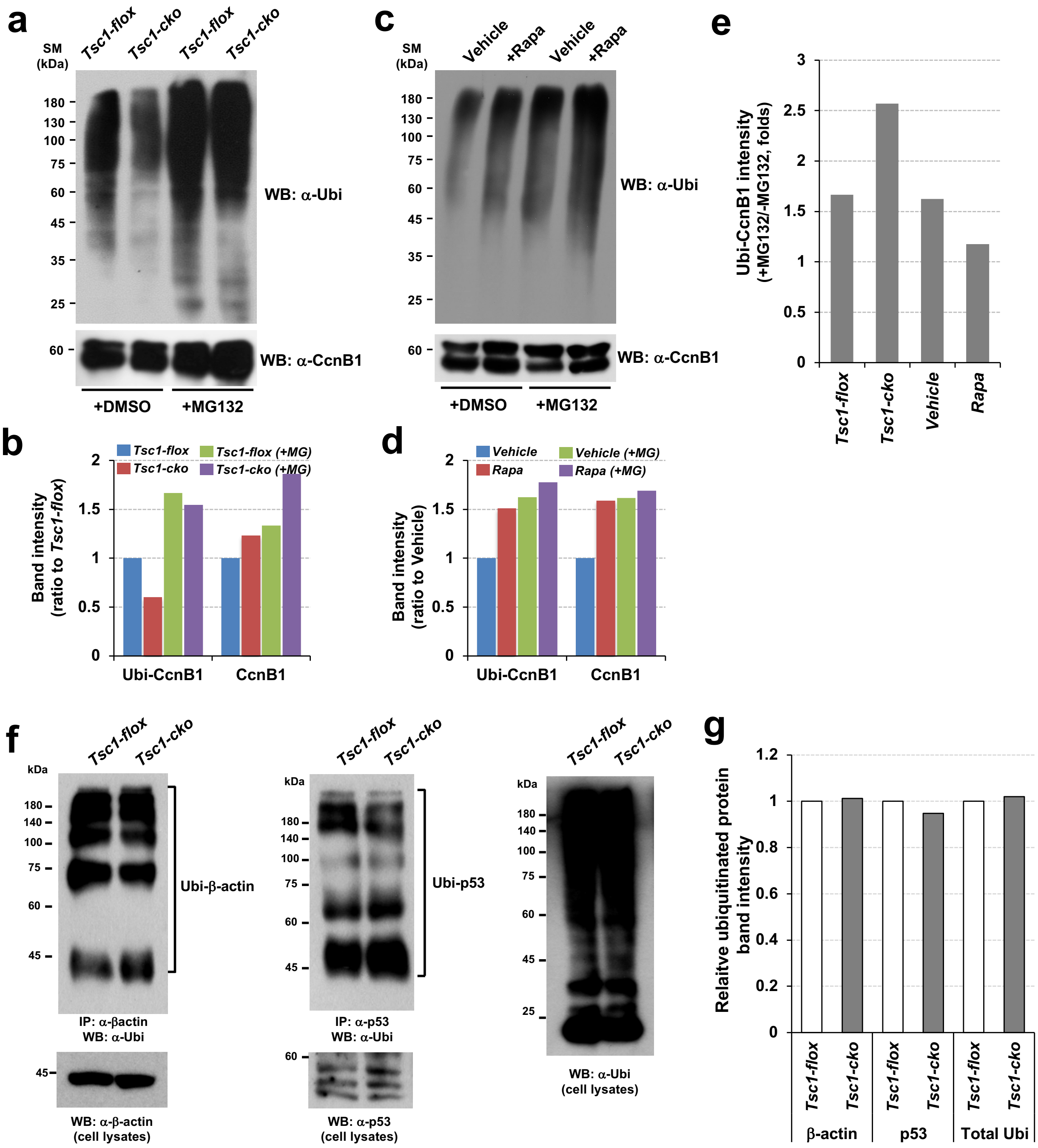

**Supplementary Figure 10. mTORC1 activation does not affect ubiquitination rate of CcnB1.** (**a**) Equal volume of vehicle (DMSO) or MG132 (15 mg/kg) was injected into the intraperitoneal space of pregnant C57BL/6J female mice, which can produce either *Tsc1-flox* or *Tsc1-cko* mice, at 14 dpc, and cell extracts were prepared from E14.5 mouse embryonic retinas (6 retinas for each sample). CcnB1 proteins were immunoprecipitated from the cell lysates by anti-CcnB1 antibody for 6% SDS-PAGE and subsequent WB analysis with anti-ubiquitin antibody to detect ubiquitinated CcnB1 (Ubi-CcnB1). Relative amounts of CcnB1 in the cell lysates subjected to the immunoprecipitation were determined by WB with anti-CcnB1 antibody. (**c**) Pregnant C57BL/6J female mice were injected with equal volume of vehicle or rapamycin (5 mg/kg) at 14 dpc in the presence or absence of MG132 (15 mg/kg). Mouse embryos were collected at 14.5 dpc from the injected mice for the detection of Ubi-CcnB1 (6 retinas for each sample). (**b** and **d**) Image pixels of Ubi-CcnB1 and CcnB1 bands in (a) and (c) were counted by *Image-J* software and relative intensities of the bands are shown in graphs. (**e**) Relative intensities of ubiquitinated CcnB1 in MG132-treated samples to those in vehicle-treated samples are also shown in a graph. (**f**) p53 and ß-actin proteins were immunoprecipitated from *Tsc1-flox* or *Tsc1-cko* mouse retinal lysates by antibodies recognizing corresponding proteins. The ubiquitinated p53 and ß-actin proteins in the immunoprecipitates were then analyzed by WB with anti-ubiquitin antibody. Ubiquitinated cellular proteins in the cell lysates were also detected by WB with anti-ubiquitin antibody. (**g**) Image pixels of bands in (f) were counted by *Image-J* software. Relative intensities of ubiquitinated proteins were compared after normalized by the intensities of non-ubiquitinated proteins.

***Supplementary Fig. 11***

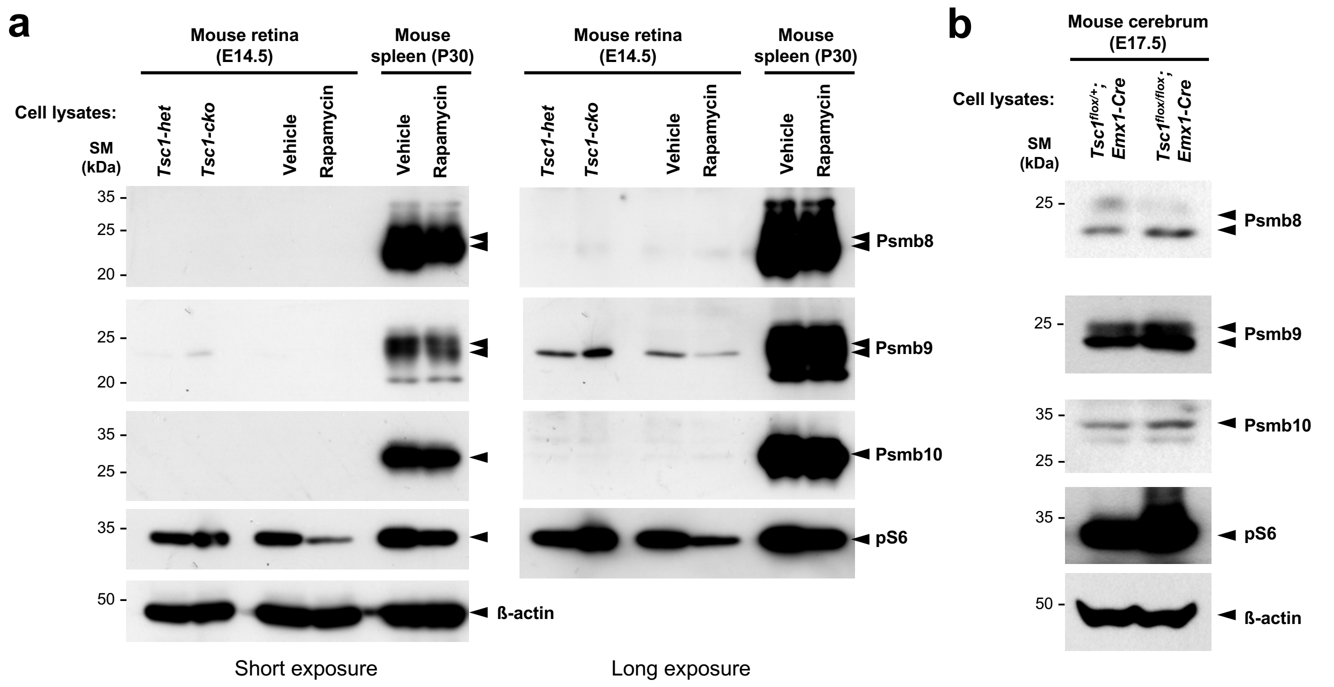


**Supplementary Figure 11. Detection of immunoproteasome subunits in mouse retina, spleen, and cerebrum having altered mTORC1 activity.** (**a**) Cell lysates (20 μg proteins/lane) were isolated from E14.5 mouse embryonic retinas and P30 mouse spleen were analyzed by 12% SDS-PAGE followed by WB analyses with corresponding antibodies. The C57BL/6J mice were injected either with vehicle or rapamycin (5 mg/kg) into their peritoneum at 16h prior to sample collection. (**b**) Cell lysates were isolated from the cerebrum of E17.5 mouse embryos with indicated genotypes and were analyzed by 12% SDS-PAGE followed by WB analyses.

***Supplementary Fig. 12***

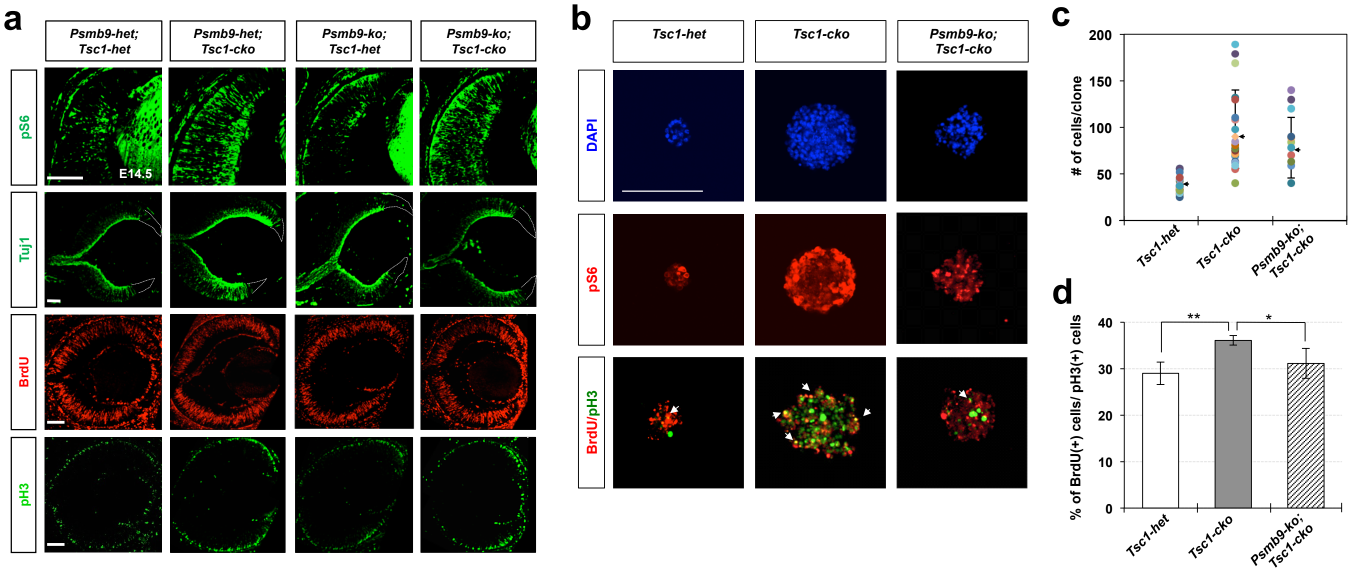


**Supplementary Figure 12. Anatomical analyses of embryonic mouse retinas having combinatorial deletions of *Psmb9* and *Tsc1*. (a)** E14.5 mouse retinal sections were stained with antibodies recognizing corresponding proteins. The areas surrounded by dotted lines in the images at second row indicate retinal area lacking Tuj1-positive post-mitotic neurons. Scale bars denote 100 μm. The quantifications of the results are shown in Fig.7b. (**b**) E13.5 *Tsc1-het*,*Tsc1-cko,* and *Tsc1-cko;Psmb9-ko* mouse retinas were dissociated into single cells and cultured to form neurospheres for 7 days as described in Supplementary Methods. The neurospheres were treated with BrdU (50 μg/ml) for 5h in prior to the fixation for immunostaining with antibodies recognizing the corresponding markers. Scale bar denotes 100μm. (**c**) Numbers of cells in individual neurospheres were obtained by counting DAPI-positive nuclei and shown in a graph. Numbers of neurosphere analyzed are 20 neurospheres for each group. Arrowheads point mean values; error bars are SD. (**d**) The numbers of pH3-positive mitotic cells among BrdU-incorporated proliferating cells were also shown in a graph. *, $p$ <0.05; **, $p$ <0.01 (ANOVA test).

***Supplementary Fig. 13***

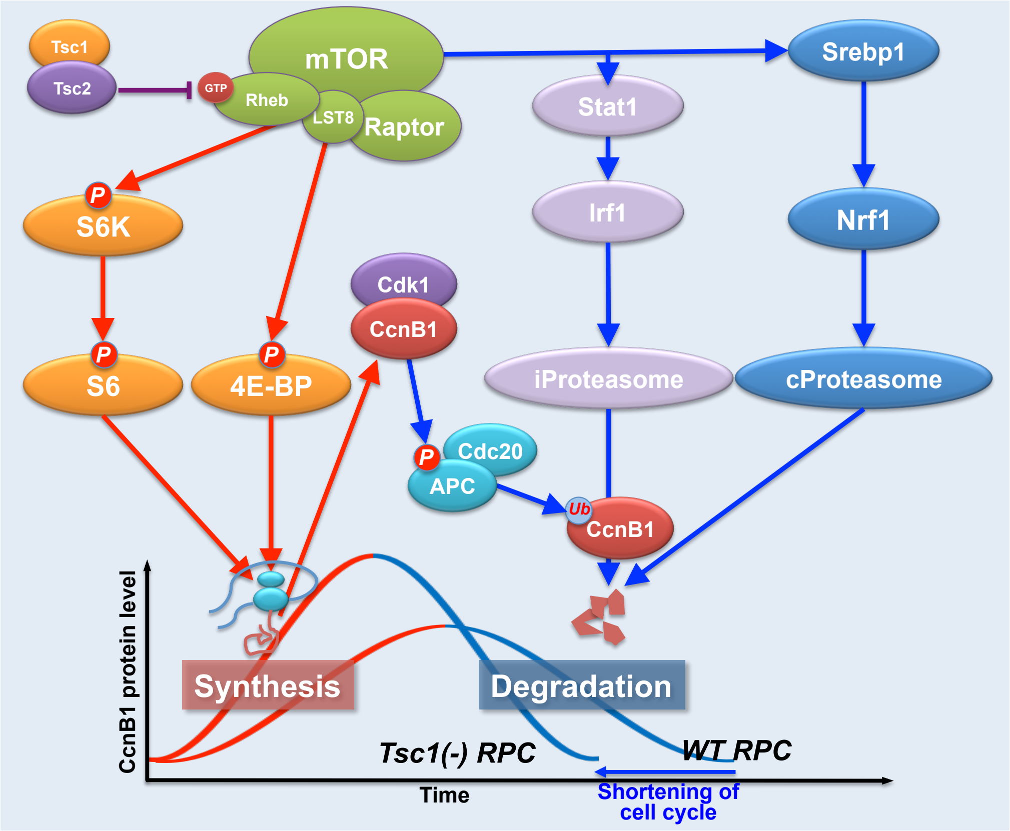


**Supplementary Figure 13. Schematic diagram depicts the roles of mTORC1 in RPC cell cycle progression.** The mTORC1, which is comprised of mTOR-LST8-Raptor, is activated by GTP-coupled Rheb upon inactivation of Tsc2 Rheb GAP. The active mTORC1 phosphorylates S6K and 4E-BP to facilitate ribosomal activity and translation initiation, respectively, and facilitates protein synthesis. Among the proteins rapidly accumulated after mTORC1 activation, CcnB1 binds to Cdk1 to induce mitotic cell cycle progression. The CcnB1 is degraded by proteasome after APC-mediated ubiquitination, allowing cells to complete mitosis and then re-enter to next rounds of cell cycle. The CcnB1 should be synthesized and degraded properly for mitotic cell cycle progression. To support the latter process, mTORC1 activates the pathways of Srebp-Nrf1 and Stat1-Irf1 to elevate cellular constitutive proteasome (cProteasome) and immunoproteasome (iProteasome) levels, respectively. This proteasomal degradation of cyclin proteins is especially important in mTORC1 hyperactive *Tsc1*-deficent RPCs, which synthesize them more rapidly than the normal. By the support of enhanced synthesis and degradation of cyclin proteins, including CcnB1 and CcnE1, *Tsc1*-deficient RPC could complete a cell cycle faster than WT RPC and produce more retinal cells ahead regular schedule.

## Supplementary Figure 14 (uncropped WB images related to Figure 1)

### Fig.1b

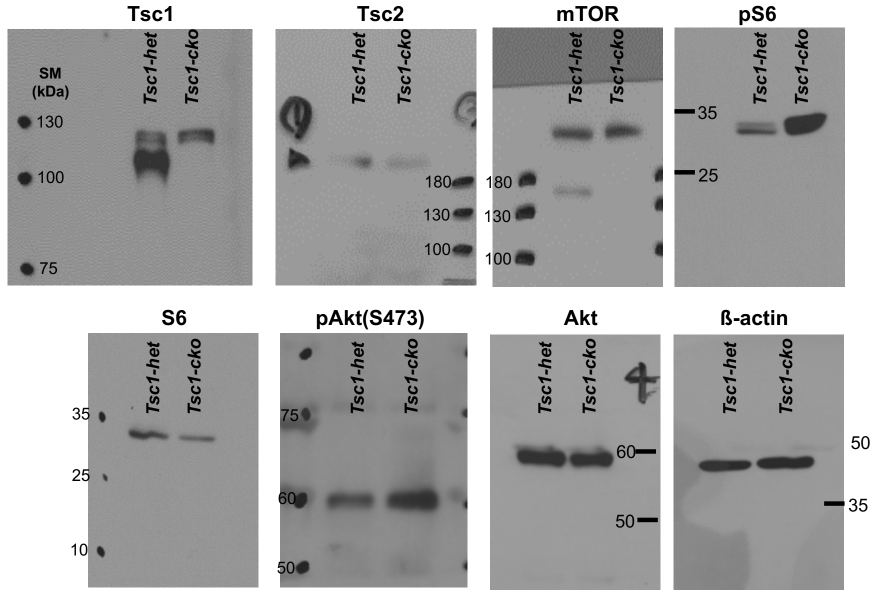

## Supplementary Figure 14 (uncropped WB images related to Figure 4)

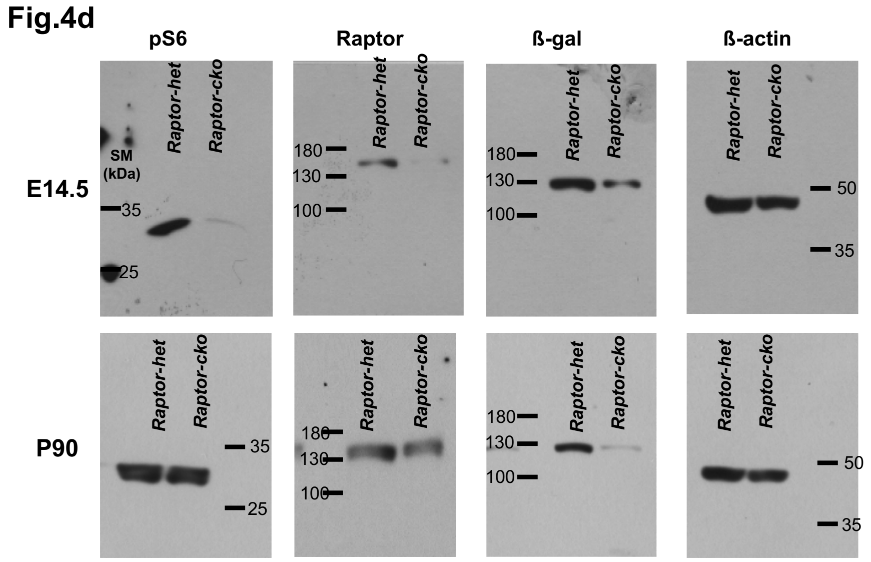

## Supplementary Figure 14 (uncropped WB images related to Figure 5)

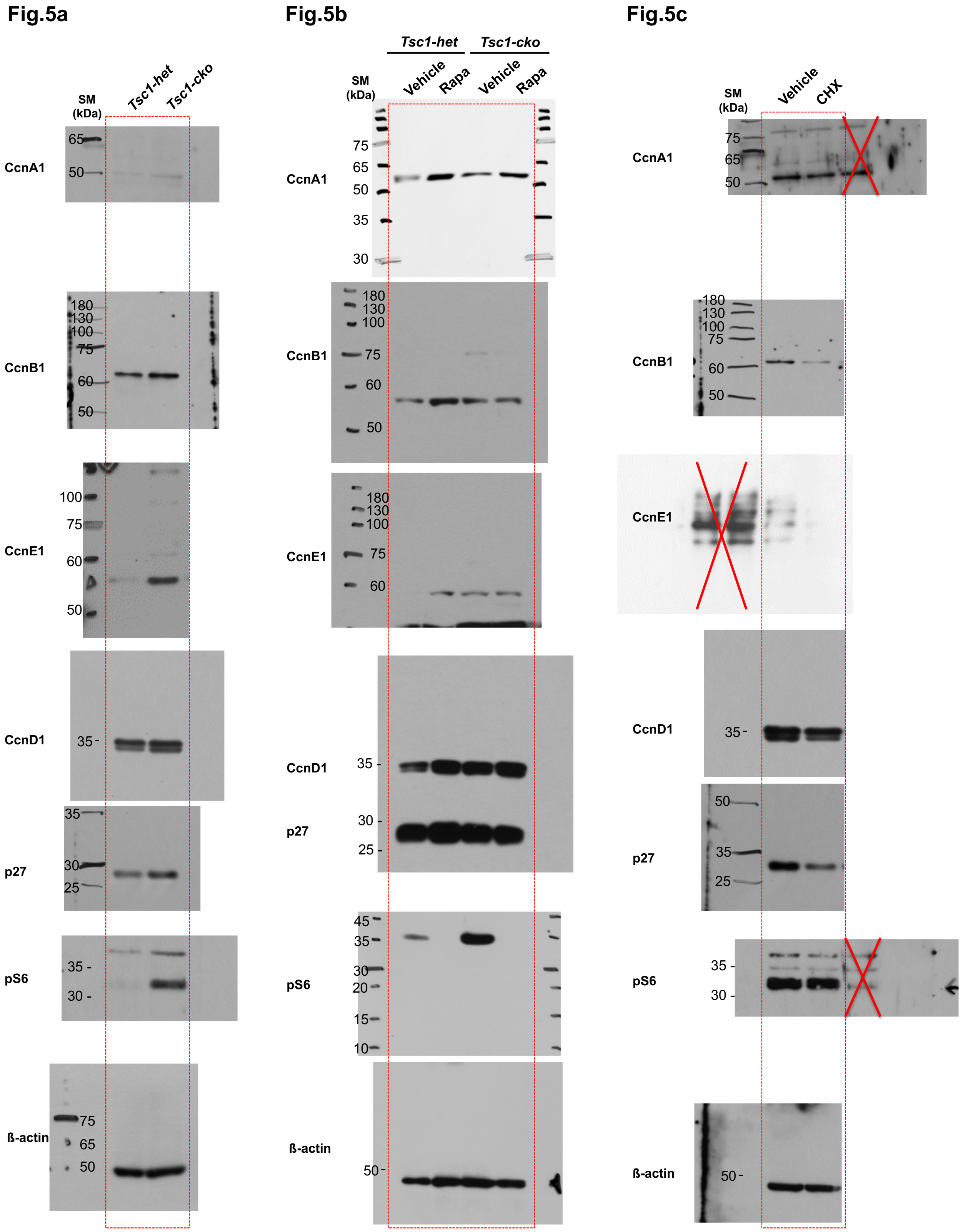

# Supplementary Figure 14 (uncropped WB images related to Figure 6)

**Fig.6c**

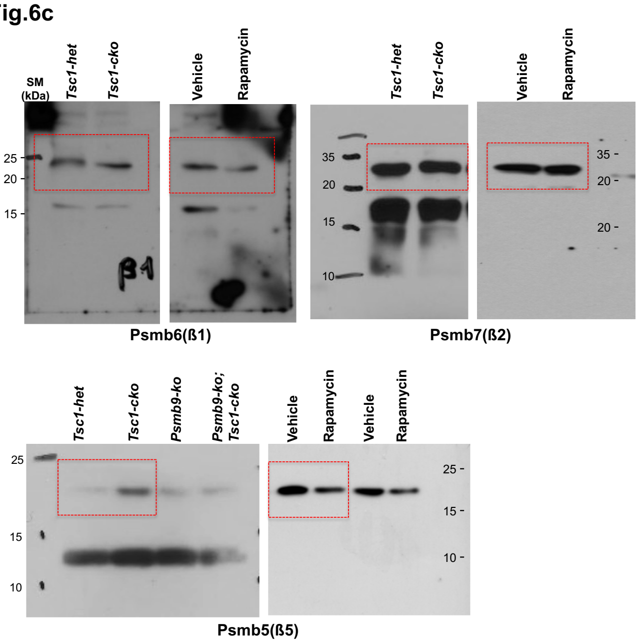


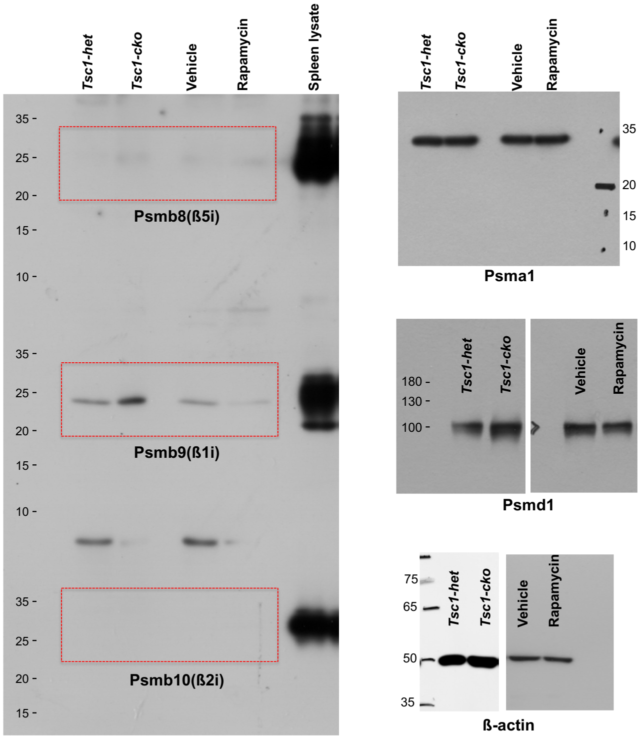


**Fig.6e**

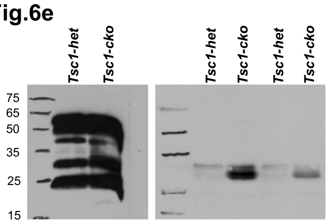

## Supplementary Figure 14 (uncropped WB images related to Figure 7)

### Fig.7d

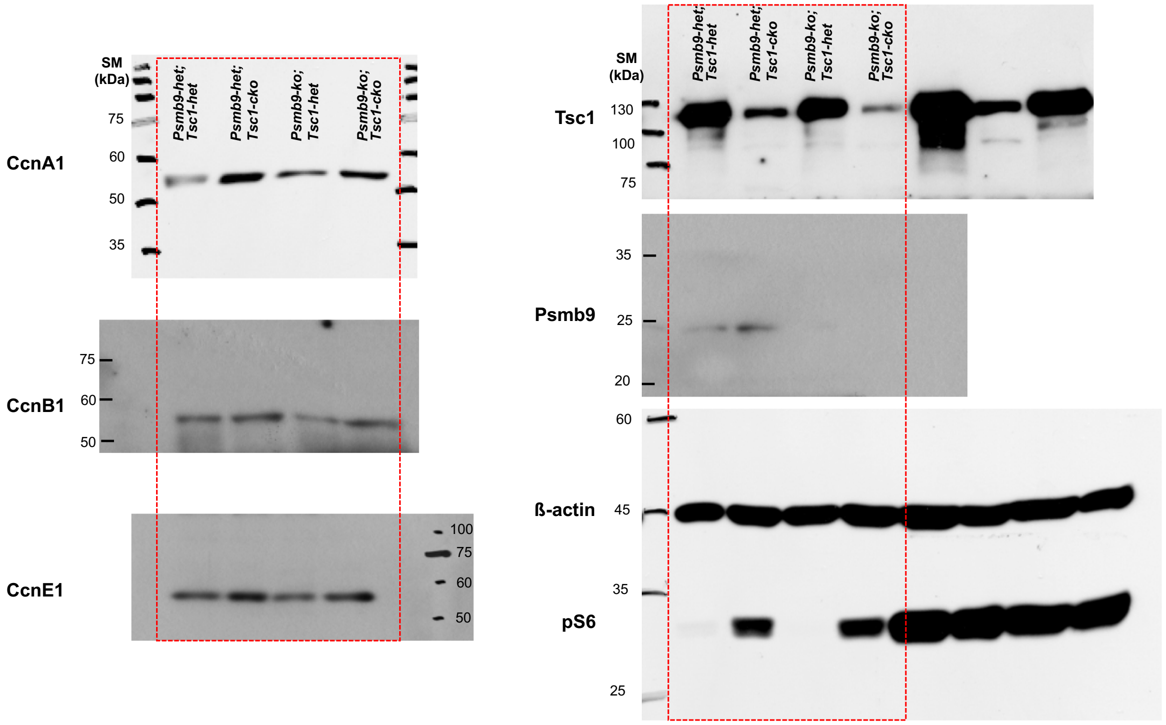

# Supplementary Figure 14 (uncropped WB images related to Figure 8)

## Fig.8b

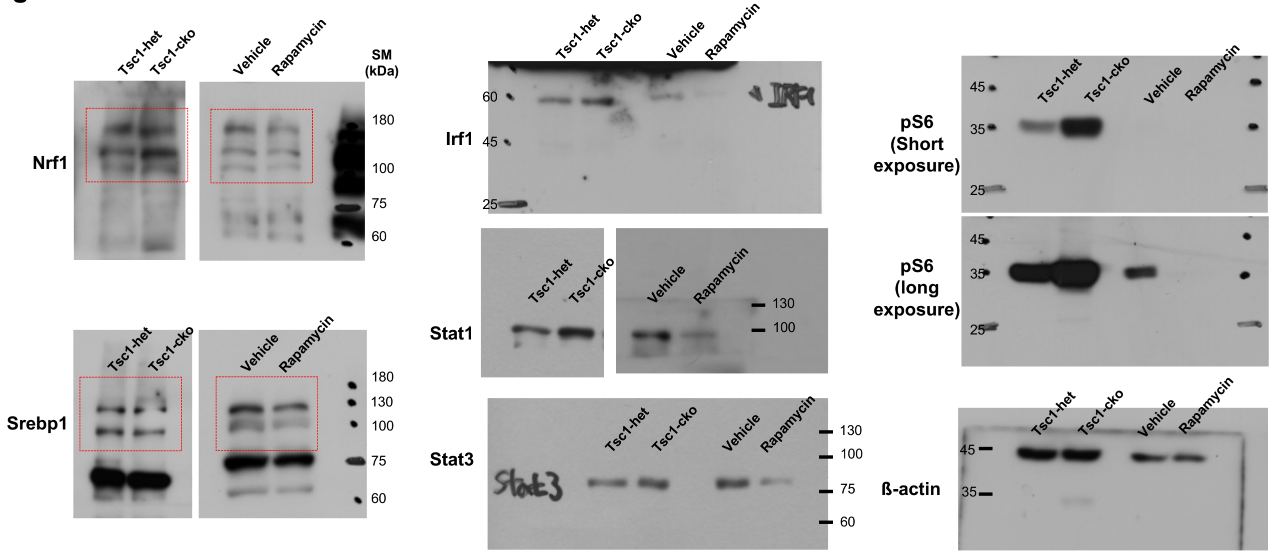


## Fig.8g

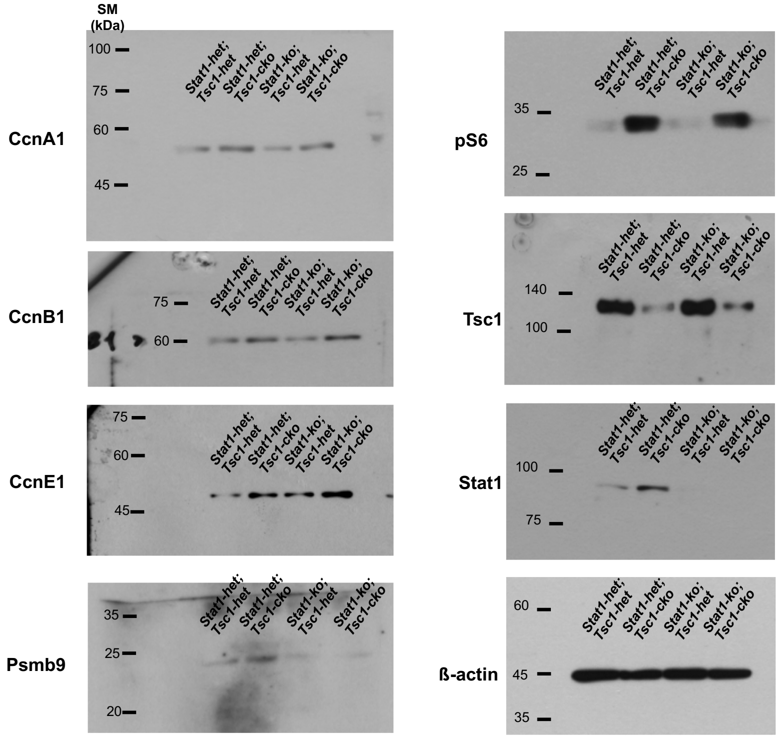

**Supplementary Figure 14 (uncropped WB images related to Supplementary Figure 1)**

## Fig.S1

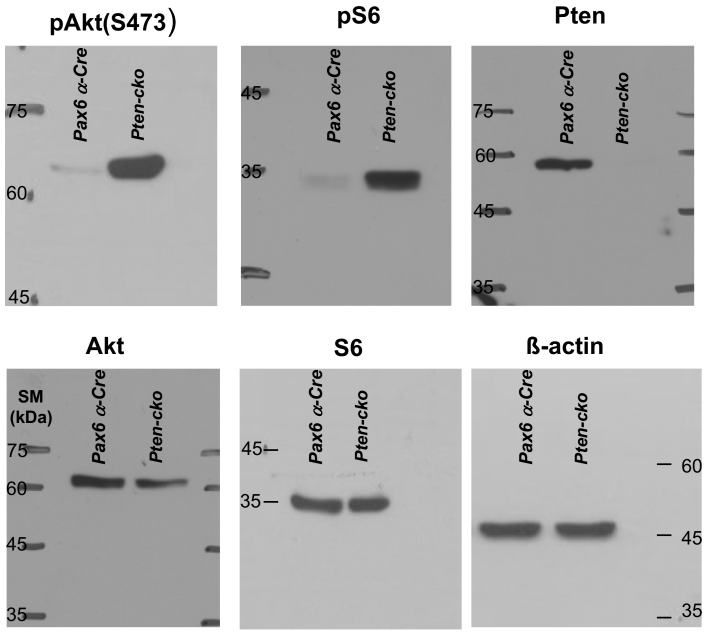

**Supplementary Figure 14 (uncropped WB images related to Supplementary Figure 5)**

## Fig.S5b

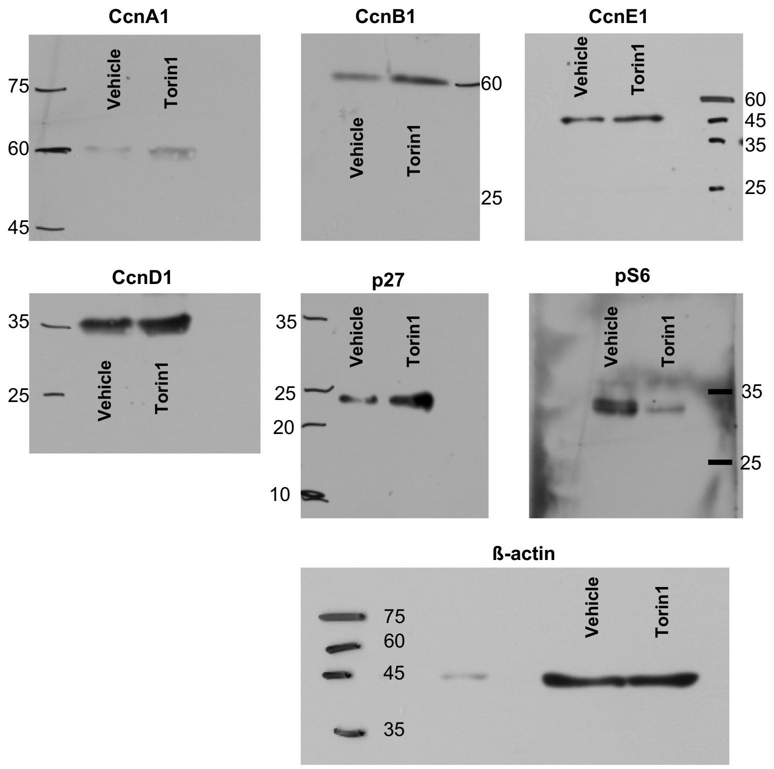

## Supplementary Figure 14 (uncropped WB images related to Supplementary Figure 11)

### Fig.S10

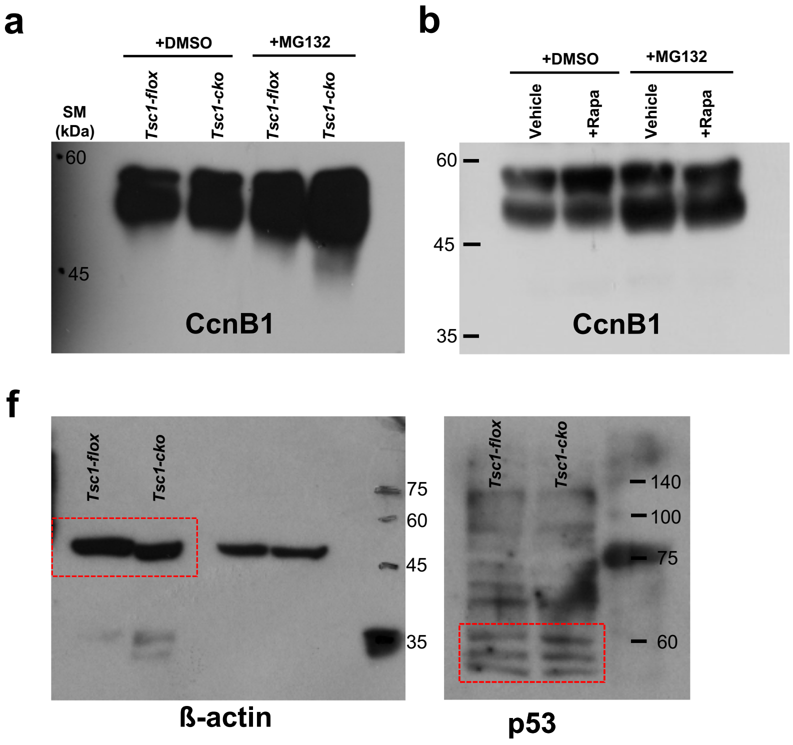

## Supplementary Figure 14 (uncropped WB images related to Supplementary Figure 11)

### Fig.S11

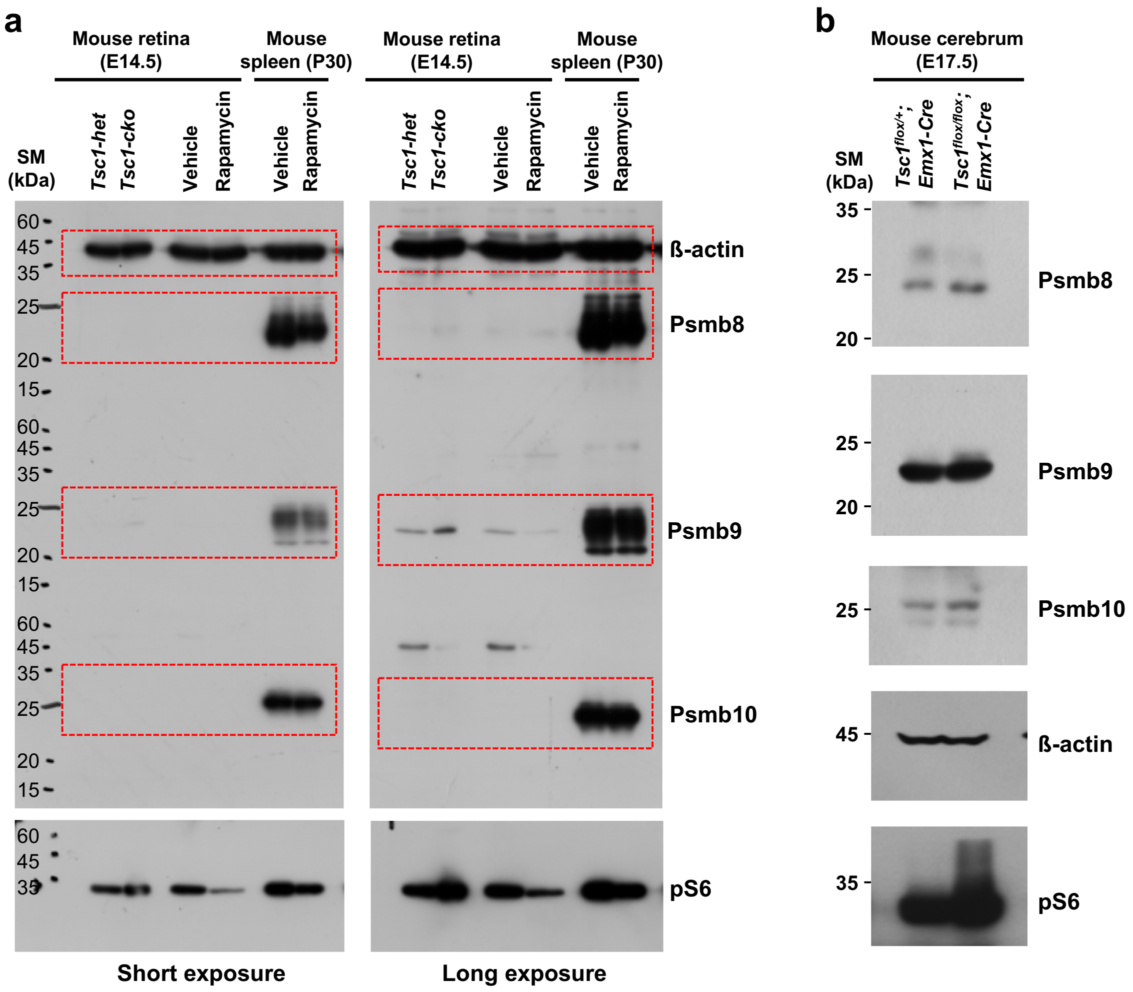

**Table S1. List of antibodies used in this study**

| Antigen | Origin | Dillution | Maker | Catalog # |
|---|---|---|---|---|
| α-tubulin | goat | 1:200 | Santa cruz biotechnology | sc-31779 |
| β-actin | rabbit | 1:1000 | Santa cruz biotechnology | sc-1616 |
| β-galactosidase | chick | WB 1:1000<br>IHC 1:200 | Abcam | ab9361 |
| 4E-BP1 | rabbit | 1:1000 | Cell Signaling Technology | 9644 |
| Akt | mouse | 1:1000 | Cell Signaling Technology | 2966 |
| BrdU(CldU) | rat | 1:200 | Novus Bio | NB500-169 |
| BrdU(IdU) | mouse | 1:200 | EXBIO | 11-286-C100 |
| Brn3b | goat | 1:200 | Santa cruz biotechnology | sc-31989 |
| Calbindin | mouse | 1:200 | Sigma | C9845 |
| Calbindin | rabbit | 1:200 | Swant | CB-38 |
| CcnA1 | rabbit | 1:1000 | Santa cruz biotechnology | sc-751 |
| CcnB1 | rabbit | 1:1000 | Abcam | ab2949 |
| CcnD1 | rabbit | 1:1000 | Santa cruz biotechnology | sc-753 |
| CcnE1 | rabbit | 1:1000 | Abcam | ab7959 |
| Crx | rabbit | 1:200 | Santa cruz biotechnology | sc30150 |
| GFP | rabbit | 1:200 | Abcam | ab290 |
| GFP | mouse | 1:200 | Santa cruz biotechnology | sc-9996 |
| Glutamine synthase (GS) | rabbit | 1:200 | Sigma | G-2781 |
| Irf1 | rabbit | 1:1000 | Cell Signaling Technology | 8478 |
| Isl1 | rabbit | 1:200 | gift from Dr. Mi-Ryoung Song | |
| Lhx3 | rabbit | 1:200 | Abcam | Ab14555 |
| M-opsin | mouse | 1:200 | Millipore | AB5405 |
| mTOR | rabbit | 1:1000 | Cell Signaling Technology | 2972 |
| Nrf1 | rabbit | 1:1000 | Santa cruz biotechnology | sc-13031 |
| Otx2 | rabbit | 1:200 | Millipore | AB9566 |
| p27(Kip1) | mouse | 1:1000 | BD | 610242 |

| | | | | |
|---|---|---|---|---|
| Pax6 | rabbit | 1:200 | Covance | PRB-278P |
| phospho 4E-BP1(T37/46) | rabbit | 1:1000 | Cell Signaling Technology | 2855 |
| phospho-Akt(S473) | rabbit | 1:1000 | Cell Signaling Technology | 4060 |
| phospho-Histone H3(S10) | rabbit | WB 1:1000<br>IHC 1:200 | Millipore | 04-1093 |
| phospho-S6(S235/236) | rabbit | WB 1:1000<br>IHC 1:200 | Cell Signaling Technology | 2211 |
| PKCα | mouse | 1:200 | Sigma | P5704 |
| Psmb10 | rabbit | 1:1000 | abcam | ab1183506 |
| Psmb5 | rabbit | 1:1000 | Enzo life science | BML-PW8895 |
| Psmb6 | mouse | 1:1000 | Enzo life science | BML-PW8140 |
| Psmb7 | mouse | 1:1000 | Enzo life science | BML-PW8145 |
| Psmb8 | mouse | 1:1000 | Enzo life science | BML-PW8845 |
| Psmb9 | mouse | WB 1:1000<br>IHC 1:200 | Santa cruz biotechnology | sc-373996 |
| Raptor | rabbit | 1:1000 | Cell Signaling Technology | 2280 |
| Rhodopsin | mouse | 1:200 | Chemicon | MAB5356 |
| S6 | mouse | WB 1:1000<br>IHC 1:200 | Cell Signaling Technology | 2317 |
| Sox9 | rabbit | 1:200 | Santa cruz biotechnology | sc-20095 |
| Srebp1 | mouse | 1:1000 | Thermo scientific | MA5-16124 |
| Stat1 | rabbit | 1:1000 | Cell Signaling Technology | 9172 |
| Syntaxin | mouse | 1:200 | Sigma | S5664 |
| Tsc1 | rabbit | 1:1000 | Cell Signaling Technology | 4906 |
| Tsc2 | rabbit | 1:1000 | Cell Signaling Technology | 3612 |
| Tuj1 | mouse | 1:200 | Covance | MMS-435P |
| Ubiquitin | mouse | 1:1000 | Cell Signaling Technology | 3936 |
| Vsx1 | mouse | 1:200 | gift from Dr. Edward Levine | |
| Vsx2 | guinia pig | 1:200 | gift from Dr. Mi-Ryoung Song | |
| Vsx2 | mouse | 1:200 | Santa cruz biotechnology | sc365519 |

**Supplementary table 2. Sequences of DNA primers used for RT-qPCR**

| Gene name | Primer direction | DNA sequence |
|---|---|---|
| β-actin | forward | 5’-TTCTTTGCAGCTCCTTCGTT-3’ |
| | reverse | 5’-ATGGAGGGGAATACAGCCC-3’ |
| CcnA1 | forward | 5’-AGTACAGGAGGACCTGTGGC-3’ |
| | reverse | 5’-ATTGACCCCATGGTCAGAGA-3’ |
| CcnB1 | forward | 5’-ACCAGAGGTGGAACTTGCTG-3’ |
| | reverse | 5’-ATTGACCCCATGGTCAGAGA-3’ |
| CcnD1 | forward | 5’-TCCTCTCCAAAATGCCAGAG-3’ |
| | reverse | 5’-GGGTGGGTTGGAAATGAAC-3’ |
| CcnE1 | forward | 5’-TTGCAAGACCCAGATGAAGA-3’ |
| | reverse | 5’-TCCACGCATGCTGAATTATC-3’ |
| Gapdh | forward | 5’- CGTCCCGTAGACAAAATGGT-3’ |
| | reverse | 5’-TTGATGGCAACAATCTCCAC-3' |
| Irf1 | forward | 5’-AATTCCAACCAAATCCCAGG-3’ |
| | reverse | 5’-AGGCATCCTTGTTGATGTCC-3’ |
| Nrf1 | forward | 5’-CCCTACTCACCCAGTCAGTATG-3’ |
| | reverse | 5’-CATCGTGCGAGGAATGAGGA-3’ |
| p27(Kip1) | forward | 5’-AGTGTCCAGGGATGAGGAAG-3’ |
| | reverse | 5’-GGGGAACCGTCTGAAACATT-3’ |
| Psma1 | forward | 5’-CCTCAGGGCAGGATTCATCAA-3’ |
| | reverse | 5’-GAGCGGCAAGCTCTGACTG-3’ |
| Psmb1 | forward | 5’-TTCCACTGCTGCTTACCGAG-3’ |
| | reverse | 5’-CGTTGAAGGCATAAGGCGAAAA-3’ |
| Psmb2 | forward | 5’-CCCAGACTATGTCCTCGTCG-3’ |
| | reverse | 5’-CCGTGTGAAGTTAGCTGCTG-3’ |

| | | |
|---|---|---|
| Psmb5 | forward | 5’-CCACAGCAGGTGCTTATATTGC-3’ |
| | reverse | 5’-GCTCATAGATTCGACACTGCC-3’ |
| Psmb6 | forward | 5’- AGGAATCATCATTGCAGGCT-3’ |
| | reverse | 5’- CGATGGCAAAGGACTGTCTTA -3’ |
| Psmb7 | forward | 5’- GCAATGGCTGTGTTTGAAGA -3’ |
| | reverse | 5’- GACCCCAAGTCATTAAAGATGC -3’ |
| Psmb8 | forward | 5’-ACTACAGTTTCTCCGCGCAA-3’ |
| | reverse | 5’-TGGGCCATCTCAATTTGAAC-3’ |
| Psmb9 | forward | 5’-GTTCCGGACGGAAGAAGTC-3’ |
| | reverse | 5’-GTTCACCACTGCTGTTCCC-3’ |
| Psmb10 | forward | 5’-CTTTACTGCCCTTGGCTCTG-3’ |
| | reverse | 5’-GTGATGGCTTCCACCAACAG-3’ |
| Psmd1 | forward | 5’-TGAATGCAGTCGTGAATGACTT-3’ |
| | reverse | 5’-GTGATAAAACACTTTCGAGGCCA-3’ |
| Srebp1 | forward | 5’-GGCTCTGGAACAGACACTGG-3’ |
| | reverse | 5’-TGGTTGTTGATGAGCTGGAG-3’ |
| Stat1 | forward | 5’-TCCCGTACAGATGTCCATGAT-3’ |
| | reverse | 5’-CTGAATATTTCCCTCCTGGG-3’ |
| Tsc1 | forward | 5’-TCTGGAGGAACACAATGCAG-3’ |
| | reverse | 5’-GACTGTATCGGGCTTGTTCTT-3’ |
